# Disease of the Gums

**Published:** 1878-09

**Authors:** 


### DISEASE OF THE GUMS.

We have just at hand a little pamphlet, consisting of a series of articles on disease of the gums, written by Dr. Geo. A. Mills, of Brooklyn, N. Y., which have been published during the past year in the Dental Cosmos. The aim in the articles contained in this pamphlet is, to present in a clear and concise manner, the principles involved in the treatment of diseased gums by Dr. Riggs who has attained some distinction in this line of Dental practice.

There are many valuable suggestions in these articles, and no one can read them carefully without deriving benefit.

We presume Dr. Mills will send the work to any one who desires it, upon sending him the postage.

